# Structural and binding characterization of the LacdiNAc-specific adhesin (LabA; HopD) exodomain from *Helicobacter pylori*

**DOI:** 10.1016/j.crstbi.2020.12.004

**Published:** 2020-12-15

**Authors:** Vasiliki Paraskevopoulou, Marianne Schimpl, Ross C. Overman, Snow Stolnik, Yajie Chen, Linh Nguyen, G. Sebastiaan Winkler, Paul Gellert, John S. Klassen, Franco H. Falcone

**Affiliations:** a School of Pharmacy, University of Nottingham, Nottingham, NG7 2RD, United Kingdom; b Structure, Biophysics and Fragment-based Lead Generation, Discovery Sciences, R&D, AstraZeneca, Cambridge, United Kingdom; c Protein Science, Discovery Sciences, R&D, AstraZeneca, Alderley Park, United Kingdom; d Alberta Glycomics Centre and Department of Chemistry, University of Alberta, Edmonton, T6G 2G2, Canada; e Innovation Strategy & External Liaison, Pharmaceutical Technology & Development, Operations, AstraZeneca, Macclesfield, United Kingdom; f Institute for Parasitology, Justus-Liebig-University Gießen, Schubertstr. 81, D-35392, Gießen, Germany

**Keywords:** *Helicobacter pylori*, LabA, HopD, Outer membrane porin, Adhesin, TEV cleavage Site, Ligand, Crystal structure

## Abstract

*Helicobacter pylori* (*H. pylori*) uses several outer membrane proteins for adhering to its host's gastric mucosa, an important step in establishing and preserving colonization. Several adhesins (SabA, BabA, HopQ) have been characterized in terms of their three-dimensional structure. A recent addition to the growing list of outer membrane porins is LabA (LacdiNAc-binding adhesin), which is thought to bind specifically to GalNAcβ1-4GlcNAc, occurring in the gastric mucosa. LabA_47-496_ protein expressed as His-tagged protein in the periplasm of *E. coli* and purified via subtractive IMAC after TEV cleavage and subsequent size exclusion chromatography, resulted in bipyramidal crystals with good diffraction properties. Here, we describe the 2.06 ​Å resolution structure of the exodomain of LabA from *H. pylori* strain J99 (PDB ID: 6GMM). Strikingly, despite the relatively low levels of sequence identity with the other three structurally characterized adhesins (20–49%), LabA shares an L-shaped fold with SabA and BabA. The ‘head’ region contains a 4 ​+ ​3 α-helix bundle, with a small insertion domain consisting of a short antiparallel beta sheet and an unstructured region, not resolved in the crystal structure. Sequence alignment of LabA from different strains shows a high level of conservation in the N- and C-termini, and identifies two main types based on the length of the insertion domain (‘crown’ region), the ‘J99-type’ (insertion ~31 ​amino acids), and the *H. pylori* ‘26695 type’ (insertion ~46 ​amino acids). Analysis of ligand binding using Native Electrospray Ionization Mass Spectrometry (ESI-MS) together with solid phase-bound, ELISA-type assays could not confirm the originally described binding of GalNAcβ1-4GlcNAc-containing oligosaccharides, in line with other recent reports, which also failed to confirm LacdiNAc binding.

## Highlights

• 2.06 Å resolution X-ray structure of LabA shares L-type fold with SabA and BabA, despite low sequence homology.
• Avoidance of multiple N-terminal truncations of LabA expressed in E. coli periplasm after redesign of expression construct.
• ESI-MS suggests very low binding affinity for lacDiNac or related carbohydrates, calling into question current ligand.
• Sequence analysis across different strains indicates 2 types of LabA (‘J99- vs 26695-type’) with high overall conservation.

## Graphical abstract

**Figure:**
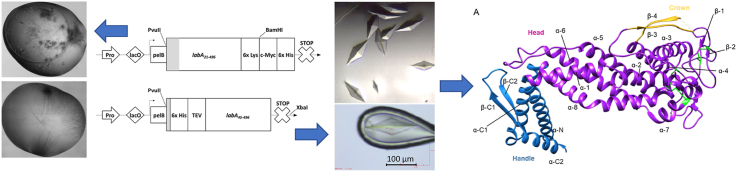

## 1 Introduction

*Helicobacter pylori (H. pylori)* is a Gram-negative microaerophilic bacterium with a strict tropism for the human gastric mucosa. *H. pylori* establishes chronic infection in the stomach despite the hostile environment with its acidic conditions and the abundance of proteolytic enzymes. As a result of this adaptation, approximately half the world's population is infected with the bacterium (Brown, 2000; Odenbreit, 2005). Among other complex adaptive mechanisms, *H. pylori*, using its helical shape and flagella, penetrates the mucus layer and adheres to the underlying gastric epithelium. The adherence is mediated by highly specific interactions between outer membrane proteins of the bacterium, called adhesins, and carbohydrate or other structures present on the surface of the epithelial cells (Salama et al., 2013).

It is estimated that *H. pylori* expresses approximately 60 outer membrane porins, of which 21 share extended sequence similarity in their N- and C- termini (Odenbreit, 2005) and at least seven are thought to mediate the attachment of the bacterium to the gastric surface epithelium (Ilver et al., 1998) (Odenbreit et al., 1999) (Peck et al., 1999) (Mahdavi et al., 2002) (Rossez et al., 2014) (Javaheri et al., 2016). The contribution of some of these proteins to bacterial adhesion and establishment of infection and chronic colonization is better understood than others.

The best characterized *H. pylori* adhesin is the Blood group antigen-binding adhesin, or BabA (Boren et al., 1993) (Ilver et al., 1998). However, not all *H. pylori* strains express BabA, and not all strains expressing BabA demonstrate affinity for Le^b^ (Hennig et al., 2004). This has been attributed to the high sequence variability observed in the extracellular domain of the protein (Nell et al., 2014). Two different groups have described the crystal structure of BabA from different *H. pylori* strains (Naim Hage et al., 2015a; Moonens et al., 2016). BabA from strain J99 is known to have affinity for Le^b^. Recently, indications of additional, previously unknown binding sites of the protein, have been reported (El-Hawiet et al., 2018).

Although BabA is thought to be responsible for the initial infection by *H. pylori* due to its affinity for Le^b^ receptors found on healthy stomach epithelium, persistent colonization of the stomach by the bacterium induces chronic inflammation, leading to the expression of sialylated glycan receptors, such as sialyl-Le^x^ and sialyl-Le^a^. The loss of Le^b^ receptors is accompanied by a loss in *babA* expression and induction of the expression of a different adhesin, sialic-acid binding adhesin or SabA, which was identified to bind the sialyl-Le^x^ receptor. This strategy allows *H. pylori* to maintain chronic infection (Magalhães and Reis, 2010; Mahdavi et al., 2002). SabA from *H. pylori* strain 26695 was the first *H. pylori* adhesin to be crystallized and structurally described (Pang et al., 2014), and later refined by Coppens and co-authors (Coppens et al., 2018).

The third structurally characterized *H. pylori* adhesin was HopQ from the strain G27 (Javaheri et al., 2016). In contrast to BabA and SabA, this adhesin does not have affinity for glycan receptors, but instead interacts with certain carcinoembryonic antigen-related cell adhesion molecules (CEACAMs). As in the previous examples, only the extracellular domain of the protein was expressed and crystallized. The crystal structure revealed similarity of the ‘head’ region among the three proteins, however HopQ lacked the prominent angle between the ‘head’ and ‘handle’ region seen in the other two, giving the exodomains of the latter two an L-shaped appearance. The ‘crown’ region, which was absent in SabA, was present in HopQ, but contained only two β-sheets instead of four as in the case of BabA. It was found that although the HopQ ‘crown’ region of the protein played a role in the binding, it did not constitute the full binding site (Javaheri et al., 2016).

While SabA, BabA and HopQ are increasingly well understood, this is not the case for other *H. pylori* adhesins. For example, we lack a complete understanding of the contribution of the paralog proteins BabB (Matteo et al., 2011) and SabB (Talarico et al., 2012) to bacterial adhesion. For some other adhesins, including LabA (Rossez et al., 2014), AlpA, AlpB (Odenbreit et al., 1999) and HopZ (Peck et al., 1999), there are no crystal structures, and in the case of AlpA, AlpB and HopZ, the receptors have not yet been identified. Generation of protein crystals and analysis of their three-dimensional conformation could prove particularly useful in the investigation of their function and the identification of ligands.

Here, we describe the crystal structure of the extracellular domain of LacdiNAc-binding adhesin, or LabA, from *H. pylori* strain J99. LacdiNAc (GalNAcβ1-4GlcNAc) is a carbohydrate structure presented on the gastric mucous cells, carried by the mucin MUC5AC, which has been suggested as a receptor for *H. pylori* adherence (Rossez et al., 2014). We also present ligand binding studies which suggest that LacdiNAc may not be the physiological ligand for this adhesin.

## 2 Material and methods

### 2.1 Materials

Oligosaccharides used in this work were as follows:

The disaccharide LacdiNAc (4-methoxyphenyl 4-O-(2-acetamido-2-deoxy-β-D-galactopyranosyl)-2-acetamido-2-deoxy-β-D-glucopyranoside, 530.52 ​Da) was purchased from Carbosynth (Compton, U.K.). Chitotriose (β-D-GlcNAc-(1 ​→ ​4)-β-D-GlcNAc-(1 ​→ ​4)-β-D-GlcNAc, MW 627.59 ​Da), chitotetraose (β-D-GlcNAc-(1 ​→ ​4)-β-D-GlcNAc-(1 ​→ ​4)-β-D-GlcNAc-(1 ​→ ​4)-β-D-GlcNAc, MW 830.79 ​Da) and chitohexaose (β-D-GlcNAc-(1 ​→ ​4)-β-D-GlcNAc-(1 ​→ ​4)-β-D-GlcNAc-(1 ​→ ​4)-β-D-GlcNAc-(1 ​→ ​4)-β-D-GlcNAc, MW 1237.17 ​Da) were purchased from Dextra (Reading, UK). HMO1 (α-L-Fuc-(1 ​→ ​2)-β-D-Gal-(1 ​→ ​4)-β-D-Glc, MW 488.17 ​Da), HMO2 (β-D-Gal-(1 ​→ ​4)-[α-L-Fuc-(1 ​→ ​3)]-β-D-Glc, MW 488.17 ​Da), HMO11 (β-D-Gal-(1 ​→ ​3)-β-D-GlcNAc-(1 ​→ ​3)-β-D-Gal-(1 ​→ ​4)-[α-L-Fuc-(1 ​→ ​3)]-β-D-Glc, MW 853.31 ​Da), HMO12 (β-D-Gal-(1 ​→ ​4)-β-D-GlcNAc-(1 ​→ ​3)-β-D-Gal-(1 ​→ ​4)[α-L-Fuc-(1 ​→ ​3)]-β-D-Glc, MW 853.31 ​Da), HMO18 (β-D-Gal-(1 ​→ ​4)-[α-L-Fuc-(1 ​→ ​3)]-β-D-GlcNAc-(1 ​→ ​3)-β-D-Gal-(1 ​→ ​4)-[α-L-Fuc-(1 ​→ ​3)]-β-D-Glc, MW 999.36 ​Da), HMO19 (β-D-Gal-(1 ​→ ​4)-β-D-GlcNAc-(1 ​→ ​3)-β-D-Gal-(1 ​→ ​4)-β-D-GlcNAc-(1 ​→ ​3)-β-D-Gal-(1 ​→ ​4)-β-D-Glc, MW 1072.38 ​Da), HMO21 (α-D-Neu5Ac-(2 ​→ ​3)-β-D-Gal-(1 ​→ ​3)-[α-L-Fuc-(1 ​→ ​4)]-β-D-GlcNAc-(1 ​→ ​3)-β-D-Gal-(1 ​→ ​4)-β-D-Glc, MW 1144.40 ​Da), HMO22 (α-L-Fuc-(1 ​→ ​2)-β-D-Gal-(1 ​→ ​3)-[α-D-Neu5Ac-(2 ​→ ​6)]-β-D-GlcNAc-(1 ​→ ​3)-β-D-Gal-(1 ​→ ​4)-β-D-Glc, MW 11440.40 ​Da), HMO25 (β-D-Gal-(1 ​→ ​3)-[α-L-Fuc-(1 ​→ ​4)]-β-D-GlcNAc-(1 ​→ ​3)-β-D-Gal-(1 ​→ ​4)-[α-L-Fuc-(1 ​→ ​3)]-β-D-GlcNAc-(1 ​→ ​3)-β-D-Gal-(1 ​→ ​4)-β-D-Glc, MW 1364.50 ​Da), HMO26 (α-D-GalNAc-(1 ​→ ​3)-[α-L-Fuc-(1 ​→ ​2)]-β-D-Gal-(1 ​→ ​4)-β-D-Glc, MW 691.25 ​Da), and HMO27 (α-D-GalNAc-(1 ​→ ​3)-[α-L-Fuc-(1 ​→ ​2)]-β-D-Gal-(1 ​→ ​3)-β-D-GlcNAc(1 ​→ ​3)-β-D-Gal-(1 ​→ ​4)-β-D-Glc, MW 1056.39 ​Da) were purchased from Elicityl SA (Crolles, France); HMO3 (α-D-Neu5Ac-(2 ​→ ​3)-β-D-Gal-(1 ​→ ​4)-β-D-Glc, MW 633.21 ​Da), HMO4 (α-D-Neu5Ac-(2 ​→ ​6)-β-D-Gal-(1 ​→ ​4)-β-D-Glc, MW 633.21 ​Da), HMO5 (α-L-Fuc-(1 ​→ ​2)-β-D-Gal-(1 ​→ ​4)-[α-L-Fuc-(1 ​→ ​3)]-β-D-Glc, MW 634.23 ​Da), HMO6 (β-D-Gal-(1 ​→ ​3)-β-D-GlcNAc-(1 ​→ ​3)-β-D-Gal-(1 ​→ ​4)-β-D-Glc, MW 707.25 ​Da), HMO7 (β-D-Gal-(1 ​→ ​4)-β-D-GlcNAc-(1 ​→ ​3)-β-D-Gal-(1 ​→ ​4)-β-D-Glc, MW 707.25 ​Da), HMO8 (α- L-Fuc-(1 ​→ ​2)-β-D-Gal-(1 ​→ ​3)-β-D-GlcNAc-(1 ​→ ​3)-β-D-Gal-(1 ​→ ​4)-β-D-Glc, MW 853.31 ​Da), HMO9 (β-D-Gal-(1 ​→ ​3)-[α-L-Fuc-(1 ​→ ​4)]-β-D-GlcNAc-(1 ​→ ​3)-β-D-Gal-(1 ​→ ​4)-β-D-Glc, MW 853.31 ​Da), HMO10 (β-D-Gal-(1 ​→ ​4)-[α-L-Fuc-(1 ​→ ​3)]-β-D-GlcNAc-(1 ​→ ​3)-β-D-Gal-(1 ​→ ​4)-β-D-Glc, MW 853.31 ​Da), HMO13 (α-D-Neu5Ac-(2 ​→ ​3)-β-D-Gal-(1 ​→ ​3)-β-D-GlcNAc-(1 ​→ ​3)-β-D-Gal-(1 ​→ ​4)-β-D-Glc, MW 998.34 ​Da), HMO14 (α-D-Neu5Ac-(2 ​→ ​6)-[β-D-Gal-(1 ​→ ​3)]-β-D-GlcNAc-(1 ​→ ​3)-β-D-Gal-(1 ​→ ​4)- β-D-Glc, MW 998.34 ​Da), HMO15 (α-D-Neu5Ac-(2 ​→ ​6)-β-D-Gal-(1 ​→ ​4)-β-D-GlcNAc-(1 ​→ ​3)-β-D-Gal-(1 ​→ ​4)-β-D-Glc, MW 998.34 ​Da), HMO16 (α-L-Fuc-(1 ​→ ​2)-β-D-Gal-(1 ​→ ​3)-[α-L-Fuc-(1 ​→ ​4)]-β-D-GlcNAc-(1 ​→ ​3)-β-D-Gal-(1 ​→ ​4)-β-D-Glc, MW 999.36 ​Da), HMO24 (β-D-Gal-(1 ​→ ​4)-[α-L-Fuc-(1 ​→ ​3)]-β-D-GlcNAc-(1 ​→ ​6)-[α-L-Fuc-(1 ​→ ​2)-β-D-Gal-(1 ​→ ​3)-β-D-GlcNAc-(1 ​→ ​3)]-β-D-Gal-(1 ​→ ​4)-β-D-Glc, MW 1144.40 ​Da), HMO32 (β-D-Gal-(1 ​→ ​4)-[α-L-Fuc-(1 ​→ ​3)]-β-D-GlcNAc-(1 ​→ ​6)-[β-D-Gal-(1 ​→ ​3)-[α-L-Fuc-(1 ​→ ​4)]-β-D-GlcNAc-(1 ​→ ​3)]-β-D-Gal-(1 ​→ ​4)-β-D-Glc, MW 1364.50 ​Da), and HMO35 (β-D-Gal-(1 ​→ ​4)--[α-L-Fuc-(1 ​→ ​3)]-β-D-GlcNAc-(1 ​→ ​6)-[α-L-Fuc-(1 ​→ ​2)-β-D-Gal-(1 ​→ ​3)-[α-L-Fuc-(1 ​→ ​4)]-β-D-GlcNAc-(1 ​→ ​3)]-β-D-Gal-(1 ​→ ​4)-β-D-Glc, MW 1510.55 ​Da) were purchased from IsoSep (Tullinge, Sweden); HMO17 (β-D-Gal-(1 ​→ ​3)-[α-L-Fuc-(1 ​→ ​4)]-β-D-GlcNAc-(1 ​→ ​3)-β-D-Gal-(1 ​→ ​4)-[α-L-Fuc-(1 ​→ ​3)]-β-D-Glc, MW 999.36 ​Da), HMO20 (β-D-Gal-(1 ​→ ​4)-β-D-GlcNAc-(1 ​→ ​6)-[β-D-Gal-(1 ​→ ​4)-β-D-GlcNAc-(1 ​→ ​3)]-β-D-Gal-(1 ​→ ​4)-β-D-Glc, MW 1072.38 ​Da), HMO28 (α-D-Neu5Ac-(2 ​→ ​3)-β-D-Gal-(1 ​→ ​4)-β-D-GlcNAc, MW 674.24 ​Da), HMO29 (α-D-Neu5Ac-(2 ​→ ​6)-β-D-Gal-(1 ​→ ​4)-β-D-GlcNAc, MW 674.24 ​Da), HMO30 (α-D-GalNAc-(1 ​→ ​3)-[α-L-Fuc-(1 ​→ ​2)]-β-D-Gal-(1 ​→ ​3)-[α-L-Fuc-(1 ​→ ​4)]-β-D-GlcNAc(1 ​→ ​3)-β-D-Gal-(1 ​→ ​4)-β-D-Glc, MW 674.24 ​Da), HMO31 (β-D-Gal-(1 ​→ ​4)-[α-L-Fuc-(1 ​→ ​3)]-β-D-GlcNAc-(1 ​→ ​6)-[β-D-Gal-(1 ​→ ​3)-β-D-GlcNAc-(1 ​→ ​3)]-β-D-Gal-(1 ​→ ​4)-β-D-Glc, MW 1218.44 ​Da), and HMO33 (β-D-Gal-(1 ​→ ​4)-[α-L-Fuc-(1 ​→ ​3)]-β-D-GlcNAc-(1 ​→ ​3)-β-D-Gal-(1 ​→ ​4)-[α-L-Fuc-(1 ​→ ​3)]-D-GlcNAc-(1 ​→ ​3)-β-D-Gal-(1 ​→ ​4)-β-D-Glc, MW 1364.50 ​Da) were purchased from Dextra (Reading, UK); HMO23 (α-D-Neu5Ac-(2 ​→ ​3)-β-D-Gal-(1 ​→ ​3)-[α-D-Neu5Ac-(2 ​→ ​6)]-β-D-GlcNAc-(1 ​→ ​3)-β-D-Gal-(1 ​→ ​4)-β-D-Glc, MW 1289.44 ​Da), and HMO34 (β-D-Gal-(1 ​→ ​3)-β-D-GlcNAc-(1 ​→ ​3)-β-D-Gal-(1 ​→ ​4)-β-D-GlcNAc-(1 ​→ ​3)-β-D-Gal-(1 ​→ ​4)-β-D-Glc, MW 1072.38 ​Da) were purchased from CarboSynth (Compton, UK). A 1.0 ​mM stock solution of each glycan was prepared in deionized water and stored at −20 ​°C until used.

### 2.2 Constructs and periplasmic protein expression

**Fig. 1:**
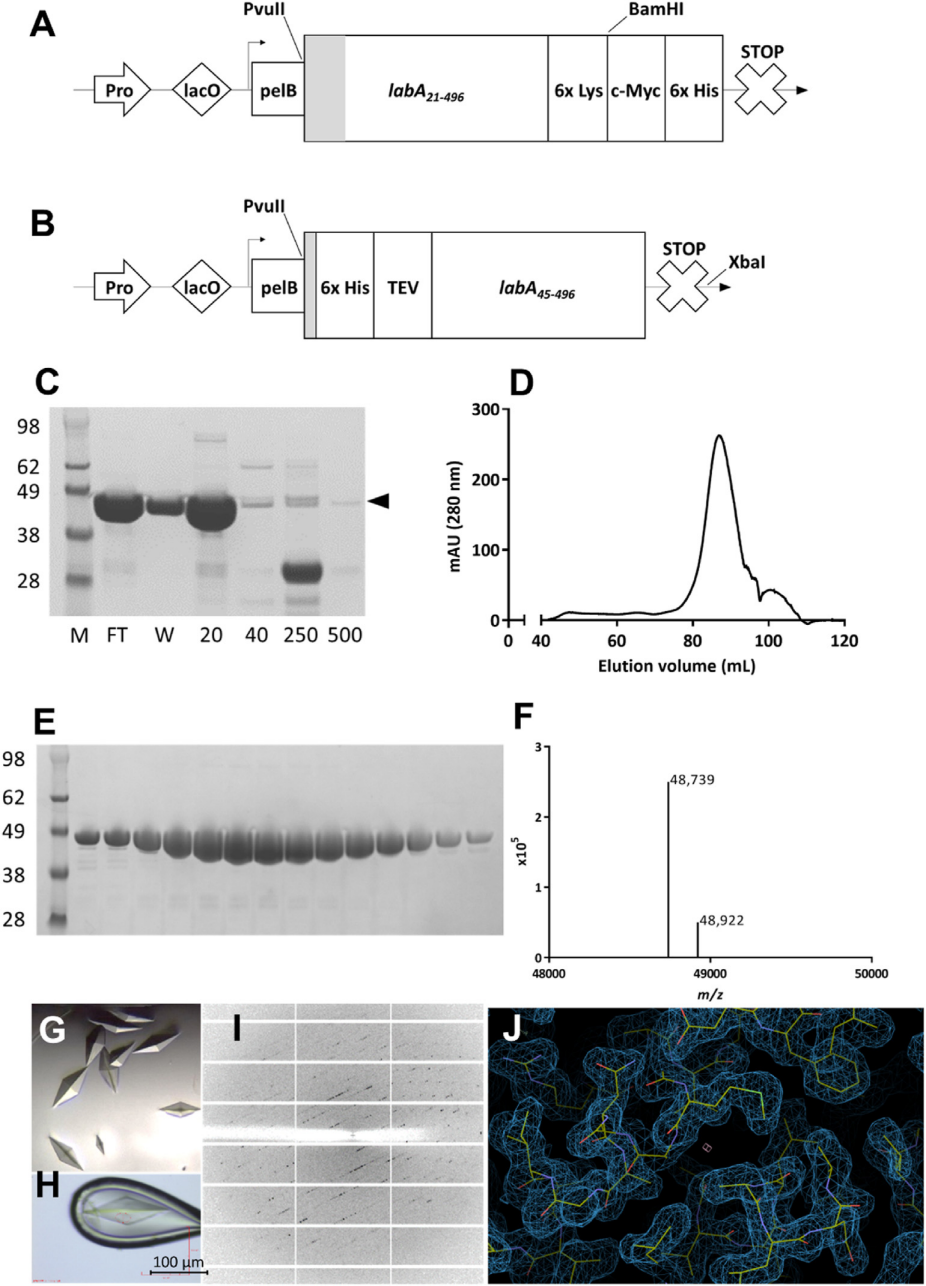
Physical map of the (A) initial and (B) improved expression cassettes for LabA_21-496_ J99 and LabA_47-496_ J99, cloned into the pOPE101 vector, using different restriction sites in each case. Highlighted in grey are the N-terminal amino acids susceptible to periplasmic truncation. The symbols stand for: Pro – synthetic P/A1/04/03 lac promoter; lacO ​– ​lac operator; arrow – start codon; pelB – cleavable leader sequence; PvuII, BamHI, XbaI ​– ​enzyme ​restriction sites; TEV ​– ​TEV cleavage site; 6x Lys – hexalysine peptide tag; c-Myc – c-Myc epitope tag; *labA*_*21-496*_ and *labA*_*47-496*_ – length of expressed genes; 6x ​His ​– ​hexahistidine peptide tag; X/STOP – stop codon. (C) Fractions from subtractive IMAC of LabA LabA_47-496_ J99 after TEV cleavage analyzed by SDS-PAGE electrophoresis. The symbols stand for: M – molecular standards, which range from 28 to 98 ​kDa; L – load; FT – flowthrough; W – wash; 20–500 – concentration of imidazole in mM. The position of the recombinant protein is indicated with a black arrowhead. (D) SEC chromatogram and (E) SEC fractions containing the protein, analyzed with SDS-PAGE and stained with InstantBlue. (F) Mass spectrum of the purified improved LabA J99_47-496_ construct, indicating the presence of one major product with a MW of 48,739 ​Da, with one minor product at 48,922 ​Da ​at a 5:1 ratio. (G) LabA J99_47-496_ bipyramidal crystals grown by sitting drop vapour diffusion in 28% poly(ethylene glycol) methyl ether 2000 and 100 ​mM Bis-Tris (pH 6.5). (H) Frozen protein crystal mounted in a nylon cryoloop during diffraction data collection. The crosshairs show the position of the X-ray beam and the oval shows the approximate beam size (31.7 ​× ​20.0 ​μm). (I) X-ray diffraction image of LabA crystals recorded at Diamond Light Source beamline I04. (J) Representative electron density map of the LabA J99_47-496_ crystal structure (PDB ID 6GMM, 2.06 ​Å resolution), showing 2F_obs_-F_calc_ map contoured at 1 σ.

The pOPE101 plasmid (PROGEN Biotechnik GmbH, Germany) construct and protocol for periplasmic expression in *E. coli* XL10-Gold which had previously been developed and optimized for BabA J99 (Naim Hage et al., 2015b) was also used for the expression of different versions of LabA J99 (Genbank Acc. No. AAD05605.1), as described in (Paraskevopoulou et al., 2019) and LabA 26695 (Genbank Acc. No. AE000511.1) (constructs see Fig. 1A and B). The following primers were used for cloning LabA_21–517K_ 26695 into pOPE101: FOR PvuII: 5′-CAGTAGCAGCTGGAAGACAACGGCTTTTTTGTG-3′ and REV (BamHI) 5′-GCTGCTGGATCCCTTCTTCTTCTTCTTCTTGAGTTCTTGACTCCTAGATTG-3’ (restriction sites underlined). The reverse primer introduces a hexalysine tag at the C-terminus of the recombinant protein. All oligonucleotide primers were from Merck, UK. The sequences of all recombinant plasmids were confirmed by Sanger sequencing (Source BioScience, Nottingham). The only modification applied was the use of 0.2 ​mM Isopropyl-β-D-thiogalactopyranoside (IPTG) for induction of protein expression, instead of 0.1 ​mM IPTG used previously. The proteins were expressed in 6 ​L of bacterial culture prior to harvesting and reconstituted with 600 ​mL of each of the two different cell lysis buffers as described in (Paraskevopoulou et al., 2019). In total, 1.2 ​L of combined periplasmic extracts were collected for each protein preparation. The redesigned construct (Fig. 1B) with a TEV cleavage site for LabA_47-496_ J99 was obtained by gene synthesis by ThermoScientific Fisher (GeneArt) (sequence available in supplementary data), and subcloned into pOPE101.

### 2.3 Chromatographic purification of recombinant protein

#### 2.3.1 Immobilized metal-ion affinity chromatography (IMAC)

The combined periplasmic extracts were incubated for a maximum of 2 ​h with 5 ​mL of Ni Sepharose 6 Fast Flow resin (Cytiva, USA) at 4 ​°C and then loaded on a gravity Econo-Column® chromatography column. The flowthrough was collected and the column was washed with ten column volumes (CV) of washing buffer, consisting of 20 ​mM Tris-Cl, pH 7.4 and 300 ​mM NaCl. Finally, the protein was consecutively eluted from the column with 3–5x CV of 20, 40, 100 and 200 ​mM and 10x CV of 500 ​mM imidazole in washing buffer. The protein content of the different fractions was analyzed with electrophoresis; the proteins were separated on NuPAGE 4–12% Bis-Tris protein gels (Thermo Fisher, USA) and the gels were stained with InstantBlue™ (Expedeon, UK).

#### 2.3.2 TEV cleavage

Approximately 30 ​mg of IMAC purified protein, at a concentration 1 ​mg/mL, were mixed with 100 ​μL of 3 ​mg/mL Tobacco Etch Virus (TEV) protease (in-house AstraZeneca product). The cleavage reaction was left to happen overnight at 4 ​°C in a dialysis setup. The reaction mixture was inserted in Spectrum™ Spectra/Por™ 1 RC dialysis membrane tubing made of regenerated cellulose dialysis with a MWCO of 6000–8000 ​Da (FisherScientific, USA), and was dialysed against 5 ​L of 20 ​mM Tris-Cl pH 7.4, 300 ​mM NaCl, in order to remove any residual imidazole from the IMAC elution fractions.

#### 2.3.3 Subtractive IMAC

The overnight reaction mixture was incubated for 1 ​h at 4 ​°C with 1 ​mL of Ni Sepharose 6 Fast Flow resin and then loaded on a gravity column. The flow through was collected and the column was washed again with 10 CV. The contents of the column were then eluted with increasing imidazole step gradient, at 20, 40, 250 and 500 ​mM imidazole. Each eluted fraction was 5 CV. The collected fractions were analyzed with electrophoresis followed by InstantBlue staining, as previously.

#### 2.3.4 Size exclusion chromatography

The purest IMAC fractions were concentrated to 5 ​mL, using a Vivaspin sample concentrator with a molecular weight cut-off of 30,000 ​Da (Merck, UK). The concentrated protein samples were loaded onto a HiLoad 16/60 Superdex 75 (120 ​mL) gel filtration column (Cytiva, USA), previously equilibrated with buffer containing 25 ​mM Bicine, pH 8.4, and 150 ​mM NaCl, connected to an ÄKTA purifier system (Cytiva, USA). The buffer was chosen based on buffer optimization results (data not shown). The flow rate was set at 1 mL/min. The fractions containing protein were analyzed with electrophoresis and InstantBlue staining, as previously.

### 2.4 Liquid chromatography – mass spectrometry

The molecular weight of purified proteins was determined by liquid chromatography (LC) – time-of-flight (ToF) mass spectrometry (MS). Approximately 5 ​μg of purified protein sample was loaded onto an Agilent 1100 Series LC (Agilent Technologies, USA) which was coupled to a time-of-flight Q-ToF Premier mass spectrometer (Waters, USA), equipped with an electron spray ionizer for acquisition in a positive ionization mode. The software MassLynx (Waters, USA) was used to analyse the data.

### 2.5 Crystallization

Protein samples were concentrated to 20 ​mg/mL and centrifuged for the removal of aggregated protein, before dispensing the crystal plates. Crystallization was performed using the sitting drop vapour diffusion method in 96-well MRC crystallization plates (Molecular Dimensions, UK) and dispensed with the assistance of the Mosquito® Robot (TTP Labtech, UK). Crystallization trials used commercial and proprietary sparse-matrix screens. Each droplet contained protein sample in 25 ​mM Bicine, pH ​= ​8.4, and 150 ​mM NaCl mixed with a precipitant solution, at a volume ratio 1:1, and was equilibrated against 50 ​μL of the precipitant solution at 4 and 20 ​°C. Bipyramidal crystals for LabA_47-496_ J99 grew in 28% poly(ethylene glycol) methyl ether 2000 and 0.1 ​M Bis-Tris (pH 6.5). Crystals appeared within one week of incubation at 20 ​°C.

### 2.6 Data collection and structure determination

Crystals were loop-mounted and briefly transferred to a drop of crystallization buffer supplemented with 20% ethylene glycol for cryo-protection, before flash-freezing in liquid nitrogen. X-ray diffraction data were collected at a temperature of 100 ​K ​at Diamond Light Source beamline I04 (wavelength 0.9795 ​Å) and were indexed and integrated using the XDS package (Kabsch, 2010). Anisotropy correction was applied to the unmerged data using the Staraniso server (Burg et al., 2008), which resulted in a resolution cut-off of 2.06 ​Å after scaling with *AIMLESS* (Evans and Murshudov, 2013). Phases were obtained by molecular replacement using the structure of BabA J99 (PDB ID 4ZH7) as a search model in the program phaser (McCoy et al., 2007). The model was completed and refined in iterative cycles of manual rebuilding in the graphics programme Coot (Emsley et al., 2010) and reciprocal space refinement using Refmac5 (Murshudov et al., 2011) and Buster (Bricogne et al., 2011). RMSD values between LabA, BabA and HopQ were determined using UCSF Chimera (v 1.14), developed by the Resource for Biocomputing, Visualization, and Informatics at the University of California, San Francisco, with support from NIH P41-GM103311. The MatchMaker function was used with a 2 ​Å cutoff for pruning and default settings (Needleman-Wunsch algorithm for alignment, BLOSUM-62 alignment matrix, and a gap extension penalty of 1).

### 2.7 Binding activity of recombinant LabA

#### 2.7.1 ELISA-type binding

For enzyme-linked immunosorbent assay (ELISA), HSA (Sigma Aldrich, USA), HSA-Le^a^, HSA-Le^b^, HSA-Le^y^, HSA-sialyl-Le^x^ (Isosep AB, Sweden) and HSA-LacdiNAc (Elicityl, France), were immobilized at a concentration of 5.0 ​μg/mL on Maxisorp plates (ThermoScientific, USA) and incubated with LabA_21-496_ and LabA_21-496_-6 ​K at a concentration range from 5 to 20 ​μg/mL. After washing off unbound glycans and proteins, the wells were sequentially incubated, with three washes between the steps, with a mouse-anti-c-Myc biotinylated antibody (AbD Serotec, USA) and the conjugate streptavidin-HRP (AbD Serotec, USA), both at a 1:2000 dilution, in order to complex with bound recombinant LabA and enable tetramethylbenzidine chromogenic detection. Absorbance was measured at 450 ​nm on a Spark® 10 ​M multimode microplate reader (Tecan, Switzerland).

#### 2.7.2 Electrospray ionization mass spectrometry (ESI-MS) affinity measurements

LabA 26695 and a single chain antibody (which served as P_ref_) were each buffer-exchanged into 200 ​mM aqueous ammonium acetate (pH 6.8) using a 10 ​kDa ​MW cutoff Amicon Ultra-4 centrifugal filter (Millipore Corp, Bedford, MA). Protein concentrations were estimated by UV absorption (280 ​nm). These stock solutions were stored at −20 ​°C until used.

All ESI-MS measurements were performed in positive ion mode using a Synapt G2 ESI-quadrupole-ion mobility separation-time-of-flight mass spectrometer (Waters UK Ltd., Manchester, UK), equipped with a nanoflow ESI source. The nanoESI tips were produced from borosilicate capillaries (1.0 ​mm o.d., 0.78 ​mm i.d.) pulled to ~5 ​μm outer-diameter using a P-1000 micropipette puller (Sutter Instruments, Novato, CA). For each measurement, approximately 10 ​μL of sample solution (containing LabA 26695, glycan and P_ref_) was loaded into the nanoESI tip. To perform ESI, a voltage of ~1 ​kV was applied to a platinum wire in contact with the solution. Mass spectra were acquired using a sampling cone voltage of 30 ​V and an extraction cone voltage of 2 ​V. The source pressure was 3.2 ​mbar and the temperature was 60 ​°C. The source wave velocity and wave height were 200 ​m ​s^−1^ and 0.2 ​V, respectively. Gas flow rates were 2 ​mL ​min^−1^ in Trap, 180 ​mL ​min^−1^ in helium cell and 90 ​mL ​min^−1^ in ion mobility cell. Ions were transmitted through the Trap and Transfer ion guides using voltages of 5 ​V and 2 ​V, respectively. At least 150 scans were collected for every acquisition. Data acquisition and processing were carried out using MassLynx (v 4.1).

Association constants (K_a_) for the interactions between LabA 26695 and the glycan ligands were quantified using the direct ESI-MS assay (Kitova et al., 2012a). Briefly, K_a_ was calculated from the abundance ratio (*R*) of the ligand-bound (PL) to free protein (P) ions, after correction for nonspecific ligand binding (El-Hawiet et al., 2012), and the initial concentrations of protein ([P]_0_) and ligand ([L]_0_), eq (1):

(1) $$K_{a}=\frac{R}{{\left[L\right]}_{0}-\frac{R}{R+1}{\left[P\right]}_{0}}$$

where R is taken to be equal to the corresponding concentration ratio ([PL]/[P]) in solution, eq (2):

(2) $$R=\frac{\sum Ab\left(PL\right)}{\sum Ab\left(P\right)}=\frac{\left[PL\right]}{\left[P\right]}$$

The reported affinities are average values from four replicate measurements performed at three different protein/glycan concentrations.

### 2.8 Data availability

The structure presented in this paper has been deposited in the Protein Data Bank (PDB) with the following codes: 6GMM. All remaining data are contained within the article or the supporting information.

## 3 Results

### 3.1 Expression and purification of LabA J99

LabA_21-496_ from *H. pylori* strain J99 was produced by periplasmic expression in *E. coli* as recently described (Paraskevopoulou et al., 2019), with C-terminal tags for solubility enhancement, immunological detection and affinity purification. The resulting protein was purified by immobilized metal-ion affinity chromatography (IMAC, Fig. S1A) and size exclusion chromatography (SEC, Figs. S1B and C). Although the IMAC and SEC protein fractions appeared pure after analysis with electrophoresis and the SEC chromatogram contained one main peak, mass spectrometry revealed multiple lengths of LabA_21-496_ J99 (Fig. S1D). The main species of 54,580 ​Da corresponds to the expected molecular weight of the protein including four cloning-derived amino acids (QVQL-) at the N-terminus of the protein. In addition, two smaller secondary products were observed; with a mass of 53,144 ​Da and 51,509 ​Da, corresponding to 9- and 24- amino acid N-terminally truncated products, respectively. Initial sparse-matrix crystallization screening yielded microcrystals in a range of different conditions. Despite optimization of the conditions and micro-seeding, the crystals remained very thin needles, and did not generate X-ray diffraction pattern for their structural analysis (Fig. S1 E and F). To obtain more suitable protein crystals, by addressing the heterogeneous processing of the N-terminal sequence during expression, a Tobacco Etch Virus (TEV)-protease cleavage site was introduced downstream of a His Tag and of the amino acids previously found to be cleaved (Fig. 1 A and B), and all C-terminal tags removed. This new variant results in a protein missing the first 46 ​amino acids (LabA_47-496_) after TEV cleavage.

Following recombinant expression, the protein was cleaved with TEV-protease. The two-step purification was repeated for the removal of the enzyme and the protein was analyzed (Fig. 1C–E). TEV and uncleaved protein were removed by subtractive IMAC, as shown in Fig. 1C. Although the SEC chromatogram of the protein sample indicated the presence of two different products (Fig. 1F), analysis of the protein fractions with SDS-PAGE revealed relatively good purity (Fig. 1E). The mass spectrum of LabA_47-496_ (Fig. 1F) revealed two products in the protein sample; the molecular mass of 48,922 ​Da is consistent with the calculated 48,760, corresponding to LabA_47-496_ with an additional N-terminal GS (Gly-Ser) added by the cloning and left after restriction with TEV (ENLYFQ∗G, cleavage site indicated by ∗). However, the ratio of the main (48,739 ​Da) product to the secondary product (48,922 ​Da) was increased and the purity of the protein sample used for protein crystallization screening was enhanced.

### 3.2 Crystallization screening of improved construct

Crystallization trials of the LabA_47-496_ construct were performed in 384 different sets of conditions in sparse-matrix screens. Within seven days, protein crystals were obtained in a crystallization drop containing 28% poly-(ethylene glycol) methyl ether 2000 and 100 ​mM Bis-Tris at a pH 6.5 and in the absence of salt, on a plate stored at 20 ​°C. The crystals displayed a bipyramidal morphology with maximum dimensions of 60 ​× ​150 ​μm (Fig. 1G) and further optimization was not required. A single crystal (Fig. 1H) was subjected to X-ray diffraction data collection (Fig. 1I) and produced a dataset to 2.06 ​Å resolution (Fig. 1J).

The crystals belonged to the space group *P*41212, with unit cell dimensions of *α* ​= ​60 ​Å, *b* ​= ​60 ​Å and *c* ​= ​265 ​Å and contained one molecule of protein per asymmetric unit. The protein structure was solved by molecular replacement, using the structure of BabA J99 as the search model, since the two amino acid sequences have 58.62% identity (53.85% for the exodomains). The crystal parameters, as well as data processing and structure refinement statistics are shown in Table 1.

**Table 1:** Crystallographic data processing and refinement statistics. Values in parentheses are for the highest resolution shell. *R*_*factor*_*= Σ*_*hkl*_*||F*_*obs*_*| - |F*_*calc*_*||/Σ*_*hkl*_*|F*_*obs*_*|.* R_free_ is the cross-validation R factor computed for the test set of 5% of unique reflections. CC_1/2_ is the Pearson correlation coefficient between the average intensities of two subsets containing randomly selected halves of the measurements for each unique reflection (Karplus and Diederichs, 2012). Ramachandran analysis was performed with MolProbity (Lovell et al., 2003) and the Z-Score was calculated by WHAT_CHECK(Hooft et al., 1996).

Protein	apo-LabA_47-496_ J99
**PDB ID**	6GMM

**Data collection**	
Space group	*P*41212

*Cell dimensions*	
*a*, *b*, *c* (Å)	59.857, 59.857, 264.744
*α, β, γ* (°)	90.0, 90.0, 90.0

Resolution (Å)	50.00–2.06 (2.09–2.06)
*R*_merge_	0.242 (1.516)
*R*_meas_	0.252 (1.590)
*R*_pim_	0.072 (0.372)
*I*/σ*I*	8.1 (1.9)
CC_1/2_	0.999 (0.599)
Completeness (%)	99.9 (98.9)
Redundancy	12.3 (11.0)

**Refinement**	
Resolution (Å)	50.00–2.06
No. reflections	384040
No. unique reflections	31169
*R*_work_/*R*_free_	0.2055/0.2518

*No. atoms*	
Protein	3229
Water	173
*B*-factors (Å^2^)	
Protein	36.59
Water	31.02

*R.m.s. deviations*	
Bond lengths (Å)	0.0101
Bond angles (°)	1.09
*Ramachandran analysis*	
Favoured	97.89% (418 residues)
Allowed	2.13% (9 residues)
Outliers	0% (0 residues)
Z-score	−0.916

**Fig. 2:**
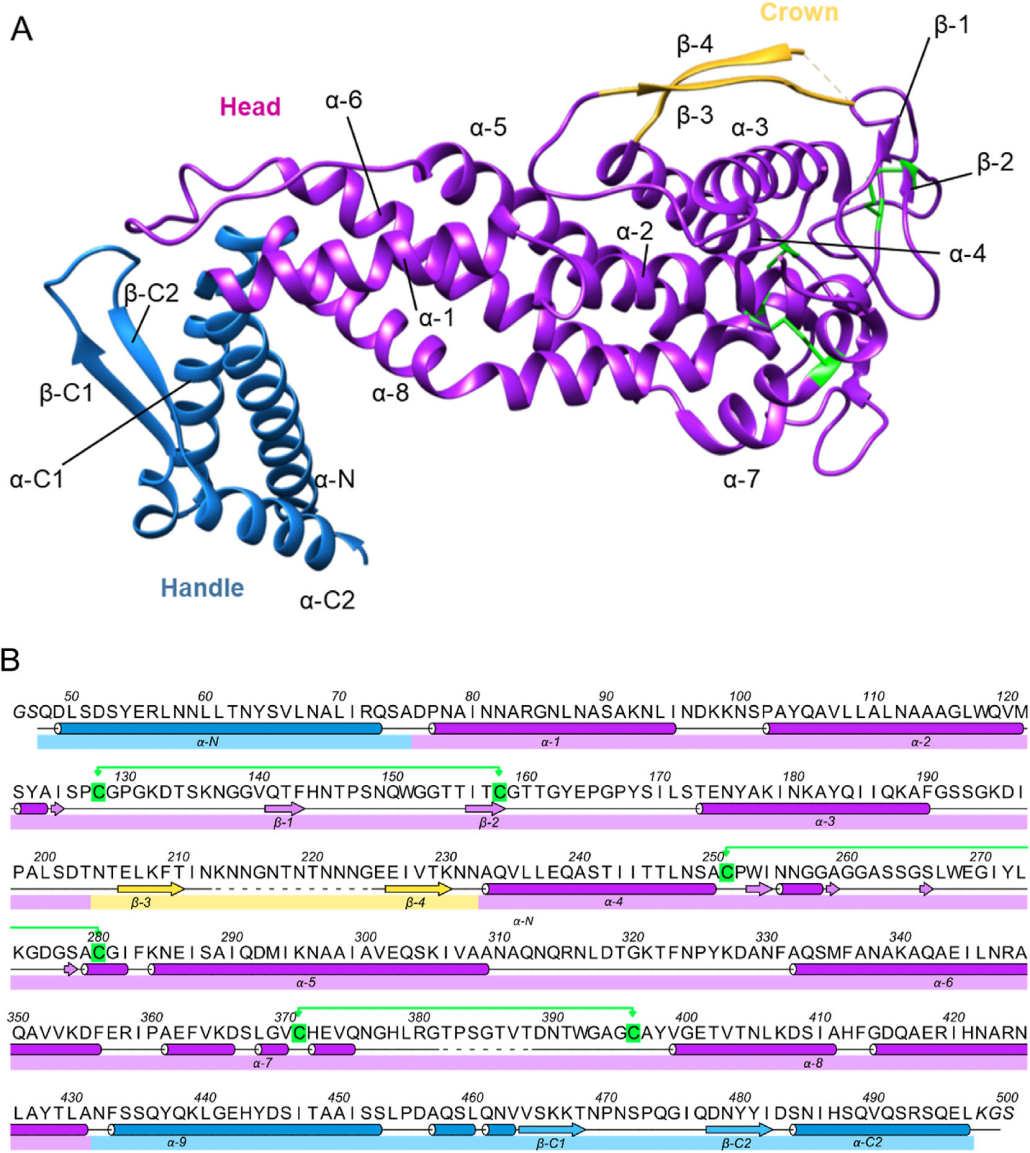
(A) Ribbon representation of the crystal structure of LabA J99_47-496_ (6GMM). Indicated are the ‘handle’ (blue), ‘head’ (purple) and ‘crown’ (gold) regions. The three disulphide bonds are coloured in green. (B) Representation of secondary structure (from crystal structure using ALINE (Bond and Schüttelkopf, 2009)). Dotted areas indicate amino acids missing from the crystal structure, coinciding with predicted areas of intrinsic disorder. Cysteines and corresponding disulphide bonds are highlighted in green. The two C-terminal amino acids (KG) are cloning-derived.

The initial model was iteratively improved by rigid body and restrained refinement interspersed with real space model building with *Coot* (Emsley et al., 2010) (Fig. 2). The final atomic model ran from residues Q47 to L496, with the addition of the two residues added downstream of the C-terminus of the protein, K497 and G498. However, there were two disordered loops not visible in the electron density map; these were between N211–E224 and G380–D388 (Fig. 2A). Both stretches are also predicted as unstructured by intrinsically disordered structure prediction algorithms, such as Globplot2.3 (Linding et al., 2003), IUPRED2 (Mészáros et al., 2018) (using ‘short disorder’ prediction type) and PONDR (Romero et al., 1997) (only N211–E224). The crystallographic model revealed that LabA J99 contains two main regions comprising mostly α-helices, called ‘handle’ and ‘head’ regions, and a smaller third region comprising two β-strands, termed the ‘crown’ region, in analogy to the structure of BabA J99 (Naim Hage et al., 2015a). The ‘handle’ region contained both the N- and C-termini of the protein; in more detail, one N-terminal (α-N) and one C-terminal (α-C1) α-helices of similar lengths formed a two-helix antiparallel coiled coil bundle. This C-terminal α-helix was followed by a two-stranded antiparallel β-sheet (β-C) before ending with a short α-helix (α-C2), which had an almost antiparallel orientation to the N-terminal α-helix. The two terminal helices protruded from the head region at right angles and were connected to the predicted transmembrane β-barrel domain. The ‘head’ region comprised a 4 ​+ ​3 α-helix bundle, also found in other *H. pylori* adhesins. Within this bundle, four α-helices (α-1, α-5, α-6 and α-8) formed an antiparallel coil bundle, similar to a tetratricopeptide repeat motif, at a near perpendicular angle to the ‘handle’ region, creating a kinked (or L-shaped) tertiary structure. The connecting features between the α-helices were: (i) A 48-amino acid segment between the α-2 and α-3 helices; this connecting segment, which extended out of the core of the head region, contained two small antiparallel β-sheet strands (β-1 and β-2); (ii) A 42-amino acid segment between the α-3 and α-4 helices; this segment contained the crown region, consisting of a two-strand antiparallel β-sheet (β-3 and β-4) between T204 and N230, and a disordered loop within the crown, between N211 and E224; (iii) A 28-amino acid loop between the α-4 and α-5 helices, with two short α-helices; (iv) A 21-amino acid loop between the α-5 and α-6 helices; (v) A 28-amino acid segment between the α-7 and α-8 helices, containing a short α-helix and a disordered loop between G380 and D388. LabA has three disulphide bonds (C127–C157, C250–C279, C370–C395), all three located in the head region (Fig. 2A).

**Fig. 3:**
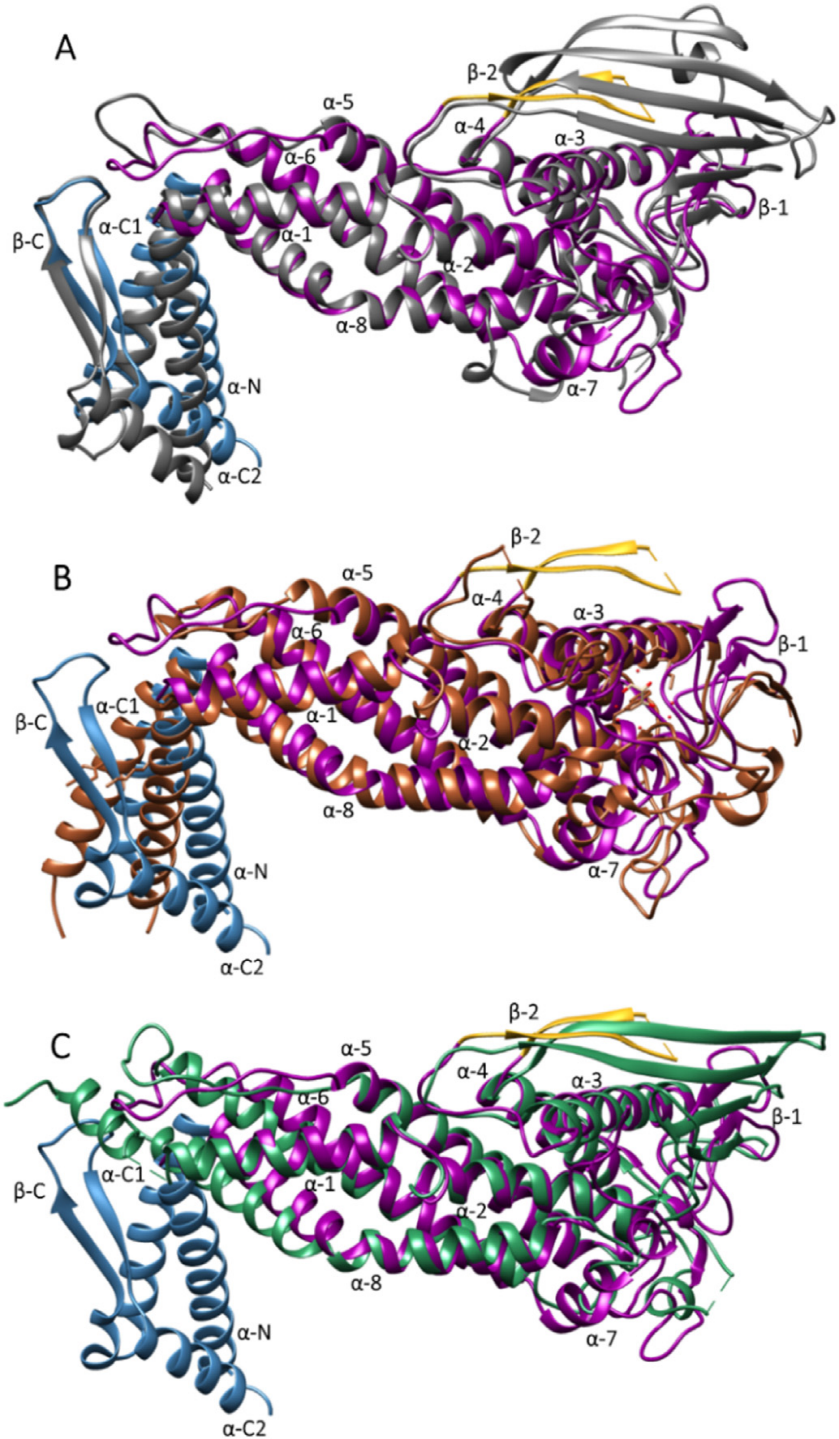
Comparison of the extracellular domain of LabA_47-496_ J99 (6GMM) with the respective domains of (A) BabA J99 (dark grey; 4ZH7, RMSD between 217 pruned atom pairs 0.921 ​Å) (B) SabA 26695 (copper; 6GW5, RMSD between 99 pruned atom pairs 1.144 ​Å) and (C) HopQ G27 (turquoise; 6AVZ; RMSD between 177 pruned atom pairs 1.054 ​Å). Highlighted in colour are the ‘handle’ (blue), ‘head’ (purple) and ‘crown’ (gold) regions of LabA_47-496_ J99.

The structure was superimposed and compared with the known structures of *H. pylori* adhesins BabA (4ZH7_A; Fig. 3A), SabA (6GW5; Fig. 3B) and HopQ (6AVZ_B; Fig. 3C), with which it shared amino acid sequence identities of 49%, 20% and 37% (exodomains only), respectively.

When comparing the full-length structures for LabA and BabA, the RMSD between 217 pruned atom pairs (cutoff used 2 ​Å) is 0.921 ​Å; without pruning, the RMSD across all 402 pairs rises to 5.493 ​Å. Using the central (‘head’) part of the molecule (ranging from S57 to V462 in BabA) results in a low RMSD of 0.874 ​Å for 208 pruned atom pairs (without pruning: 4.959 ​Å). The pruned atoms in the ‘head’ region correspond to the inserted ‘crown’ region (G170-D257, RMSD 16.832 ​Å), as the crown is much smaller and contains an unstructured region in LabA. Without pruning, the RMSD for AA 57–462 rises to 4.780 ​Å. Matching the handle regions D27-P56 and V461–K528 gives RMSD values of 0.465 and 1.147 ​Å, respectively. Overall, these results show that the handle and head regions are very similar between LabA and BabA, but are connected at slightly different angles, with the largest discrepancies between the two structures found in the crown region.

Despite the relatively low sequence identity, all these proteins share high conformational similarity in their head regions, where a 4 ​+ ​3 α-helix bundle was present; however, significant differences were observed in the crown region, which may be related to each protein's ligand binding specificity. The ‘crown’ regions of LabA and HopQ (called ‘insertion domain’ in (Bonsor et al., 2018)) comprises a short and long β-sheet, respectively, while a crown structure is altogether absent in SabA. Differences were also observed in the handle region of the adhesins. The original structure of SabA 26695 (4O5J) lacked the β-sheet (β-C) present in LabA and BabA, as the C-terminal last 61 ​amino acids residues were not visible in the structure (Pang et al., 2014). The more recent structure of SabA J99 (6GW5) by Coppens et al. (2018) however includes a short hairpin (residues 393–438) matching the longer β-C domain in BabA J99 (4ZH0).

### 3.3 ELISA-type and ESI-MS analysis of ligand binding by recombinant LabA

**Fig. 4:**
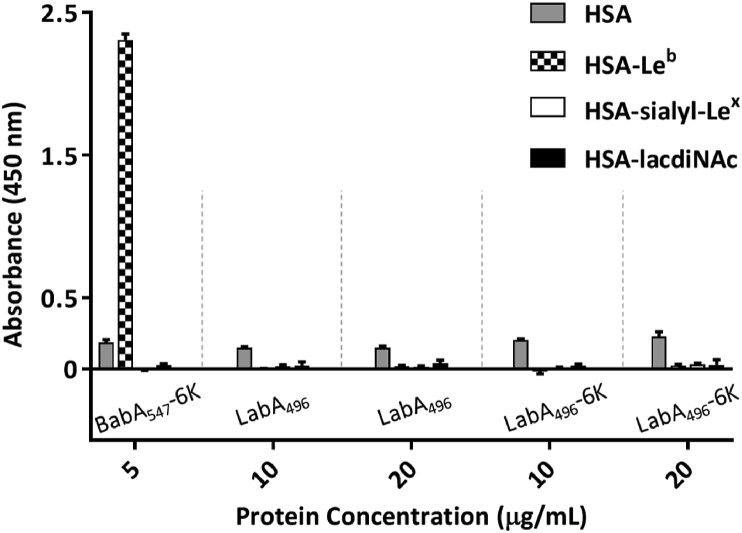
Lack of LabA binding to LacdiNAc in sandwich ELISA-type format. Binding of LabA_21-496_ and LabA_21-496_-6 ​K, in 20 ​mM Tris-Cl pH 7.4 and 300 ​mM NaCl, to HSA and HSA-conjugated known *H. pylori* ligands: HSA-Lewis^b^, HSA-sialyl-Lewis^x^ and HSA-LacdiNAc, was assessed using two different concentrations of LabA (10 and 20 ​μg/mL). BabA_547_-6 ​K at 5 ​μg/mL, in the same buffer solution, was used as a positive control (n ​= ​3, error bars represent the standard error of the mean).

Rossez et al. (2014) indicated that LabA is a LacdiNAc-specific adhesin, which mediates the adhesion of *H. pylori* to human gastric mucins. To verify this binding ability, a sandwich-type ELISA was carried out in order to test the binding of the recombinantly expressed LabA_21-496_ and LabA_21-496_-6 ​K (both from J99 strain; the 6 ​K-variant has an additional hexalysine tag see Fig. 1A) to LacdiNAc and the ligands that are well known to be *H. pylori* receptors, Lewis^b^ and sialylated-Lewis^x^. However, in contrast to BabA, which was used as positive control, neither of the two proteins showed binding to any of the ligands, at concentrations up to 20 ​μg/mL (Fig. 4).

Native Electrospray Ionization Mass Spectrometry (ESI-MS) was initially performed on aqueous ammonium acetate (50 ​mM, pH 6.8, 25 ​°C) solutions containing LabA_21-496_ 26695 (4.6 ​μM) in the absence and presence of LacdiNAc. A reference protein (P_ref_, 0.4 ​μM) was added to the latter solution to correct the mass spectrum for the occurrence of nonspecific ligand-protein binding during the ESI process (Wang et al., 2003). However, due to the presence of multiple N-terminally truncated forms of LabA 26695 occurring in different amounts, interpretation of data was difficult (data not shown). Because of the difficulty with interpreting binding to multiple truncated forms of LabA_21-496_ 26695, the new variant LabA_47-496_, equivalent to that which had enabled crystallization, was examined.

**Fig. 5:**
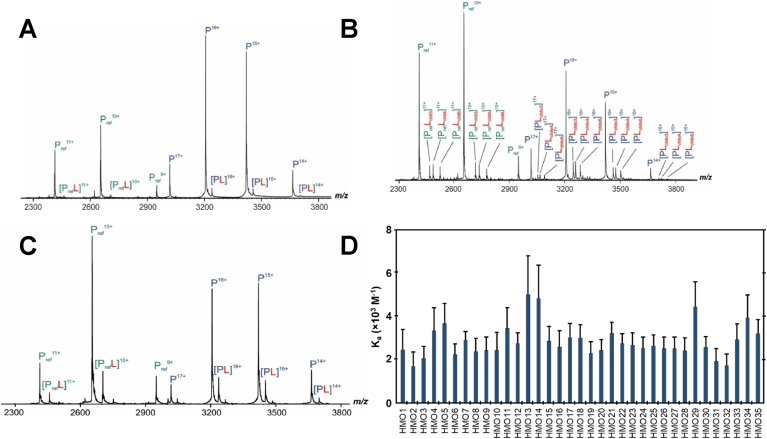
(A) Binding of LabA_47-496_ 26695 to LacdiNAc measured by ESI-MS. The representative ESI mass spectrum was acquired in positive ion mode for a 100 ​mM aqueous ammonium acetate solution (pH 6.8, 25 ​°C) of LabA_47-496_ 26695 (P, 2.5 ​μM), LacdiNAc (L, 15 ​μM) and P_ref_ (3.2 ​μM). (B) Binding of LabA_47-496_ 26695 to chitotriose, chitotetraose and chitohexaose measured by ESI-MS. The representative ESI mass spectrum was acquired in positive ion mode for a 100 ​mM aqueous ammonium acetate solution (pH 6.8, 25 ​°C) of LabA_47-496_ 26695 (P, 2.5 ​μM), chitotriose (L_chit3_, 48 ​μM), chitotetraose (L_chit4_, 48 ​μM) and chitohexaose (L_chit6_, 48 ​μM) and P_ref_ (3.2 ​μM). (C) LabA binding to HMOs measured by ESI-MS. The representative ESI-MS mass spectrum was acquired in positive mode for a 100 ​mM aqueous ammonium acetate solution (pH 6.8, 25 ​°C) of LabA47-496 26695 (P, 5 ​μM), HMO1 (L, 50 ​μM) and P_ref_ (3.2 ​μM). (D) Summary of the affinities (K_a_) of 35 HMOs (HMO1 ¬– HMO35) for LabA_47-496_ 26695 measured by ESI-MS.

Binding of this newly produced LabA_47-496_ 26695 with LacdiNAc was measured. Fig. 5A shows a mass spectrum of an aqueous ammonium acetate (100 ​mM, pH 6.8, 25 ​°C) solution of LabA_47-496_ 26695 (2.5 ​μM), P_ref_ (3.2 ​μM) and LacdiNAc (15 ​μM). Only one isoform of LabA was detected, with a MW of 51,277 ​Da. After correction of nonspecific binding using the reference protein method (Kitova et al., 2012b; Sun et al., 2006), the affinity of LacdiNAc for LabA was found to be 3088 ​± ​252 ​M^−1^ (mean ​± ​s.d.). We next decided to investigate binding to chito-oligosaccharides due to the structural similarities to LacdiNAc. The affinities of chitotriose, chitotetraose and chitohexaose (structures see Fig. S2) were also measured. Shown in Fig. 5B is a mass spectrum obtained from an aqueous ammonium acetate (100 ​mM, pH 6.8, 25 ​°C) solution with LabA 26695 (2.5 ​μM), P_ref_ (3.2 ​μM) and three chito-glycans (each 48 ​μM). The affinities for the three oligosaccharides ranged from 1000 ​M^−1^ to 2000 M^−1^.

Having established only weak binding affinity of LabA_47-496_ 26695 and no binding at all of LabA_21-496_ J99 in ELISA-like format (Fig. 4) for LacdiNAc, we next decided to screen a set of 35 human milk oligosaccharides (HMO1-HMO35, see Table S1), all of which contain Lac and the majority contain LacNAc, in an attempt to identify potential binders with higher affinity (for schematic structures, see Fig. S2). A representative example for HMO1 is shown in Fig. 5C, and the results of this screening are summarized in Fig. 5D and Table S1. Notably, LabA was found to bind all 35 HMOs, although with uniformly weak affinity (ranging from 1700 to 5000 ​M^−1^).

## 4 Discussion

While our initial attempts to crystallize LabA protein were unsuccessful, removal of the C-terminal tags and addition of an N-terminal TEV cleavage site resulted in suitable crystals. It has been reported that excessive solubility can play a role in making protein crystallization more challenging. In particular, highly soluble protein versions bearing a C-terminal pentalysine tag have been found to yield small needle-shaped crystals, similar to those obtained by us here (Fig. 1S) inappropriate for X-ray diffraction, even when high protein concentrations were achieved (Islam et al., 2015). Homogeneity of the recombinant protein was improved by introducing a TEV cleavage site in the N-terminus. However, truncation within the TEV cleavage site by periplasmic proteases had to be prevented; for this reason, the TEV cleavage site was introduced after the first nine amino acids in the N-terminus of the protein, which was the main area of non-specific proteolytic cleavage during periplasmic expression.

At the same time, limiting the proportion of unstructured regions in the protein was also considered during the design of the new expression construct. It was known from the crystal structure of BabA J99 that the three C-terminal tags, used for the enhancement of solubility, detection by Western Blotting and purification with IMAC, were not visible in the crystal structure. This confirmed the conformational flexibility of these sequences. We removed the C-terminal hexalysine and c-Myc-tags and moved the hexahistidine tag needed for IMAC to the N-terminus, upstream of the TEV cleavage site. This approach resulted in the new recombinant LabA variant which yielded protein crystals sufficient for X-ray crystallography during initial screening already, without the requirement for further optimization.

Overall, the protein adopts a similar structure to the previously described adhesins of *H. pylori*.

Despite low amino acid sequence similarity, all four crystallized *H. pylori* adhesins (LabA, BabA, SabA, and to a lesser extent HopQ) adopt a three-dimensional L-shape, suggesting that this structure may fulfil some yet to be discovered functional role. The highest similarity of the LabA amino acid sequence to that of BabA was corroborated by the superimposition of their crystal structures and the obtained low RMSD values. The similarity among all adhesins was most pronounced in the handle and head and particularly low in the crown region, where the glycan binding site of BabA is found. While this explains the lack of binding affinity of LabA for Le^b^, it does not provide any information about the actual binding site of this protein. In the absence of a ligand, the crown appears partially disordered in most adhesin structures.

A multiple alignment of HopD/LabA sequences from 84 different strains shows that LabA is highly conserved at both N- and C-termini, with the exception of a central region (amino acids 201–226) (Fig. S4) in which most of the inter-strain variability is found. This region overlaps with the unstructured region in our J99 LabA (N211–E224) and coincides with the position of the insertion domains in BabA and HopQ. With regards to this variable region, HopD/LabA sequences appear to fall into two separate categories, the *H. pylori* ‘J99-type’, with an overall total length of approx. 686–691 amino acids and a shorter insertion domain of approx. 31 ​amino acids, and the *H. pylori* ‘26695 type’, with an approximate total length of 702–711 amino acids and a longer insertion domain of approx. 46 ​amino acids. In the absence of a ligand co-crystal structure, the exact significance of these structural variations between strains remains unclear. While BabA also shows strain variation in the crown region/insertion domain, the variations in this region are stronger in BabA, which has led to the identification of two hypervariable/diversity loops ((N Hage et al., 2015) (Moonens et al., 2016)). In contrast, in LabA, there is almost no variation within the ‘J99-type’ or ‘26695-type’ insertion domains. It is interesting to note in this context that in the original paper by Rossez and coauthors (Rossez et al., 2014) two strains suggested to bind to LacdiNac (B128 and 26695) had the longer insertion domain (’26695 type’), while the J99 strain, with the shorter insertion domain, also were thought to bind to LacdiNAc, as suggested by the strong inhibition in the presence of 0.5 ​mM soluble disaccharide. This would appear to suggest that the ability to bind to LacdiNAc is not related to the type of insertion domain present in LabA, or that the shared properties of short vs. longer insertion domains are sufficient to mediate ligand binding.

However, our ESI-MS screening indicated that LabA only weakly binds to Lac/LacNAc-containing oligosaccharides, without any clear specificity. Furthermore, we were not able to detect any binding to LacdiNAc-HSA in an ELISA-type assay. We do not believe that the omission of the first 46 ​N-terminal amino acids in the improved construct can be responsible for the lack of LacdiNAc binding for two reasons. First, the longer LabA_21-427_ variant tested in ELISA-type format also did not bind to the putative ligand. The first 20 ​amino acids correspond to the signal peptide. Secondly, as known from the existing crystal structures of BabA (4ZH7) and SabA (6GW5, 4O5J), and in line with the obtained structure for LabA (6GMM), the N-terminal region is located close to the membrane insertion domain, which is buried deep in the outer membrane of *H. pylori*, and is a very unlikely binding site, as it would not be able to access its putative ligand on epithelial cells.

Because of our inability to detect any binding to LacdiNAc, we extended our study to the binding of 35 HMOs with similar structures and to three chito-oligosaccharides of different lengths (Fig. 5). The latter only differ from LacDiNAc in the axial vs. equatorial orientation of the hydroxy group in the C4 position (Fig. S2). Binding of LabA to all the tested oligosaccharides was invariably found to be non-specific and of low affinity (K_a_ values summarized in Table S1).

This lack of binding to LacdiNAc is in good agreement with the recent observation by Mthembu and co-workers (Mthembu et al., 2020). The authors of this recent study were not able to detect any binding of *H. pylori* J99 or 26695 strain (or 15 other strains tested, representing different world populations) using a variety of methods, including whole bacteria binding assays in microtiter plates. This was not due to a lack of expression or translation of LabA protein, as this could be identified by LC-MS/MS. The complete lack of binding of *H. pylori* bacteria to LacdiNAc, together with our inability to detect specific and high affinity binding of the recombinant LabA protein, suggests that LacdiNAc might not be the physiological ligand for this adhesin, and that the glycan preference of LabA remains to be elucidated. Alternatively, the function of LabA in promoting binding may be accessory, rather than direct, supporting the structure and/or binding activity of another adhesin with specificity for LacdiNAc as determined by Rossez et al. (2014), or specific for another epithelial ligand, as suggested by the work from Mthembu and co-workers (Mthembu et al., 2020). From this point of view, reintroducing the original term HopD for this protein instead of LabA is an option worth considering.

## Funding and additional information

This research has been supported through joint funding from 10.13039/501100000266EPSRC (Grant EP/L01646X), 10.13039/100004325AstraZeneca R&D and the 10.13039/501100000837University of Nottingham. Y.C., L.N. and J.S.K. acknowledge funding from the 10.13039/501100003178Alberta Glycomics Centre. Ross Overman is currently employed by Leaf Expression Systems. FHF is currently fully funded by a grant from the LOEWE DRUID (Novel Drug Targets against Poverty-Related and Neglected Tropical Infectious Diseases).

## CRediT authorship contribution statement

**Vasiliki Paraskevopoulou:** Investigation, Methodology, Validation, Writing - original draft, Writing - review & editing. **Marianne Schimpl:** Investigation, Formal analysis, Methodology, Validation, Writing - review & editing. **Ross C. Overman:** Methodology, Conceptualization, Writing - review & editing. **Snow Stolnik:** Funding acquisition, Supervision, Writing - review & editing. **Yajie Chen:** Investigation, Methodology, Writing - review & editing. **Linh Nguyen:** Investigation, Methodology, Writing - review & editing. **G. Sebastiaan Winkler:** Funding acquisition, Supervision, Writing - review & editing. **Paul Gellert:** Methodology, Conceptualization, Writing - review & editing, Funding acquisition, Supervision. **John S. Klassen:** Conceptualization, Methodology, Writing - review & editing. **Franco H. Falcone:** Funding acquisition, Project administration, Supervision, Writing - original draft, Writing - review & editing.

## Declaration of competing interest

The authors declare that they have no known competing financial interests or personal relationships that could have appeared to influence the work reported in this paper.

## Glossary

6K: hexalysine tag
CEACAM: carcinoembryonic antigen-related cell adhesion molecule
BabA: Blood group antigen-binding adhesin
CV: column volume
Da: Dalton
DTT: Dithiothreitol
EDTA: ethylenediaminetetraacetic acid
ELISA: enzyme-linked immunosorbent assay
ESI-MS: electrospray ionization mass spectrometry
HSA: human serum albumin
Hop: *Helicobacter* outer membrane protein
IMAC: immobilized metal-ion affinity chromatography
IPTG: isopropyl β-D-1-thiogalactopyranoside
LabA: LacdiNAc-binding adhesin
LB: lysogeny broth
LC-ToF-MS: liquid chromatography time-of-flight mass spectrometry
PDB: protein data bank
PTFE: polytetrafluoroethylene
RSMD: root mean square deviation of atomic positions
SDS-PAGE: sodium dodecyl sulphate polyacrylamide gel electrophoresis
SEC: size exclusion chromatography
TEV: Tobacco Etch Virus
