# Supplementary material for: Structural and binding characterization of the LacdiNAc-specific adhesin (LabA; HopD) exodomain from Helicobacter pylori
Source: Curr Res Struct Biol. 2020 Dec 15;3:19–29. doi: 10.1016/j.crstbi.2020.12.004 (PMC8244420; doi:10.1016/j.crstbi.2020.12.004)
Supplement: Supplementary file 1 [file mmc1.docx]

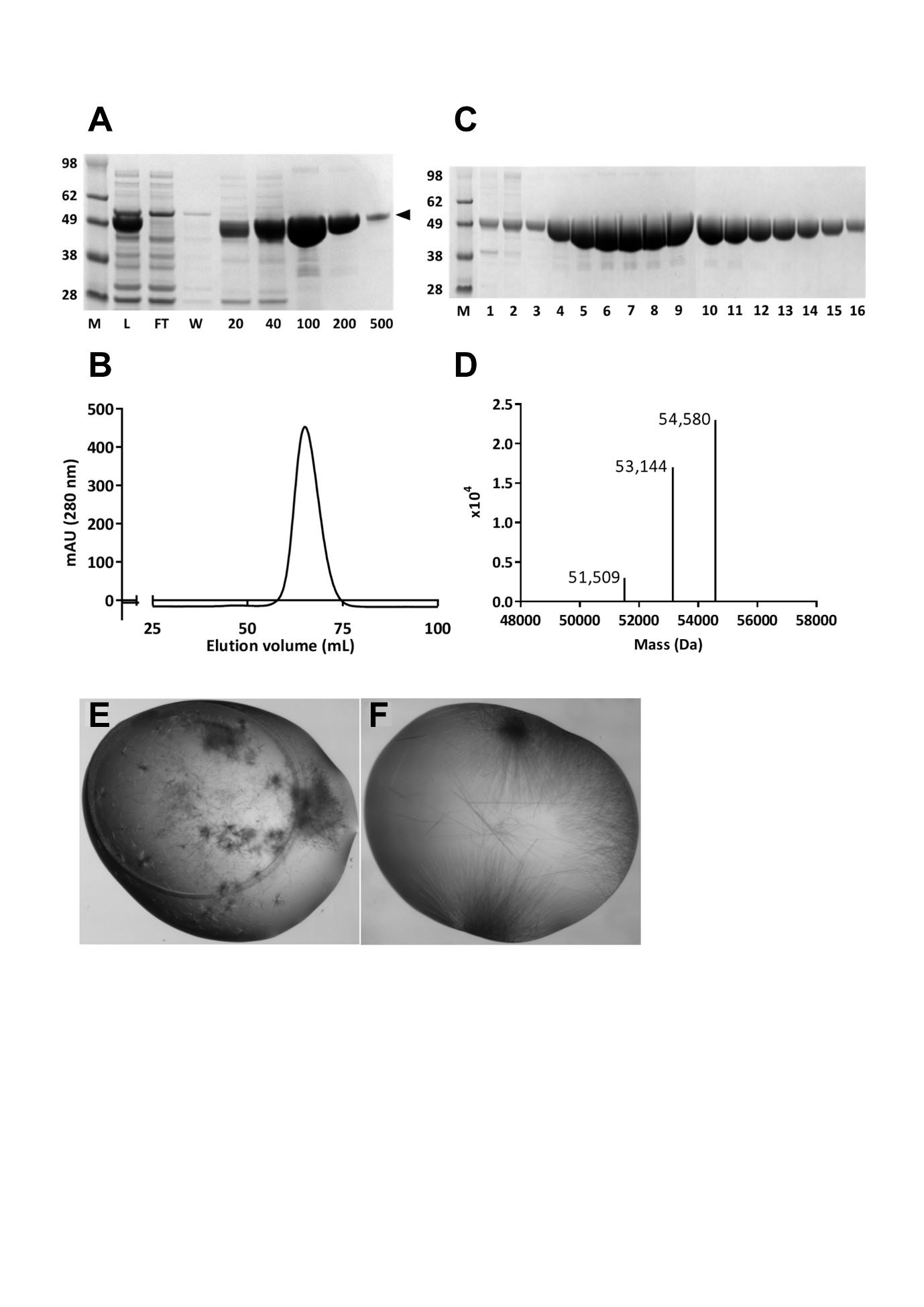


**Figure S1.** (A) IMAC fractions of the original LabA_21-496_ J99 analysed with electrophoresis. The symbols stand for: M – molecular standards, which range from 28 to 98 kDa; L – load; FT – flowthrough; W – wash; 20-500 – concentration of imidazole in mM. The recombinant protein position is highlighted with the black arrowhead. (B) SEC chromatogram and (C) SEC fractions containing recombinant protein, analysed with SDS-PAGE and stained with InstantBlue. (D) Mass spectrum of the purified original LabA_21-496_ J99 construct. (E) Optical microscope images of protein microcrystals used as seeds to encourage crystal growth of (F) bigger protein crystals. Only long and thin, needle-shaped crystals of the original LabA_21-496_ J99 were obtained, unsuitable for X-ray crystallography.


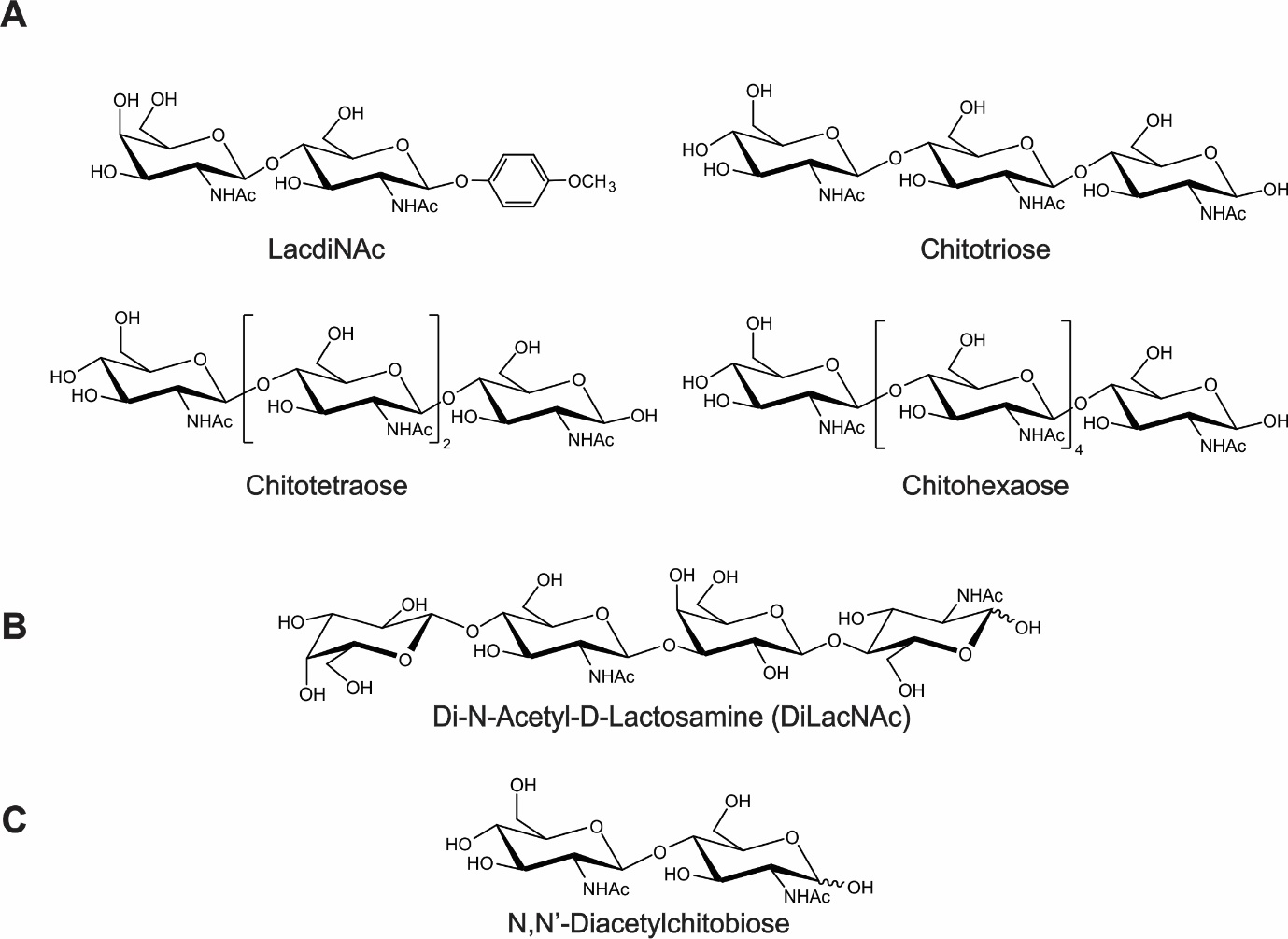


**Figure S2*.*** Structures of some the candidate ligands used in this study: (A) Structure of LacdiNAc, the motif reported to be recognized by LabA (shown here as the commercially available 4-methoxyphenyl derivative). (B) DilacNAc is a tetrasaccharide, dimer of a modified LacdiNAc, where the galactosamine residue has been replaced with a galactose residue. (C) N, N’-diacetylchitobiose is a dimer of acetylated glucosamine residues, instead of a disaccharide consisting of an acetylated galactosamine and an acetylated glucosamine, which is LacdiNAc.


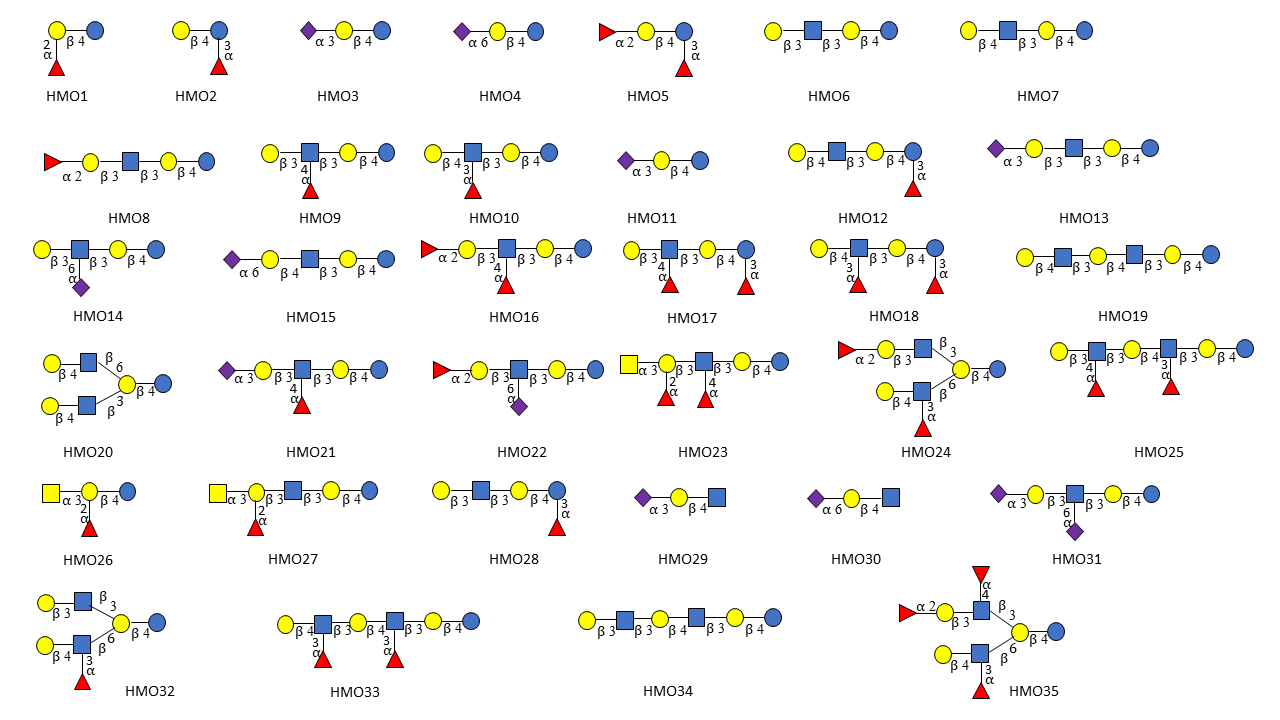


**Figure S3.** Structures of the 35 HMOs screened for binding to LabA_47-496_ 26695 in this study.


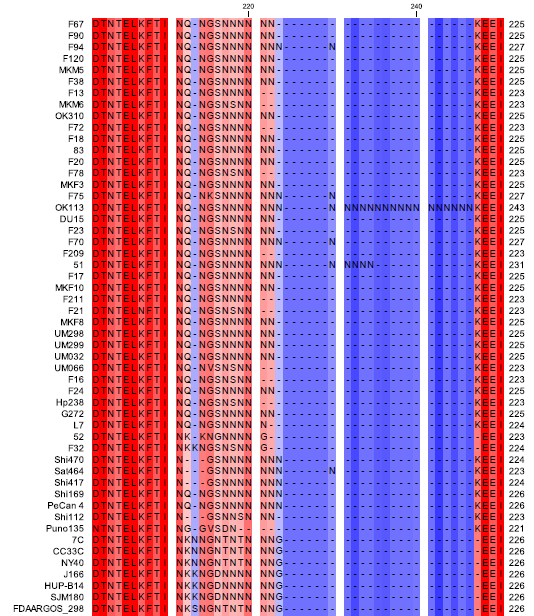

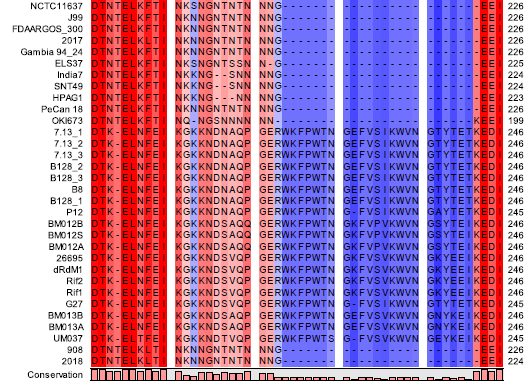


**Figure S4. Conservation of the putative crown region of LabA from different H. pylori strains.** Multiple sequence alignment of the predicted as crown sequence of LabA from 84 different H. pylori strains revealed two main types of putative crowns; in particular, the majority of the strains expressed a version of LabA bearing the shorter crown-length (“Type J99”, line 54; black arrowhead), while fewer strains expressed a version of LabA with a longer putative crown amino acid sequence (“Type 26695”, line 75; green arrowhead). Alignment obtained using CLC Main Workbench (Qiagen).

**Table S1.** Summary of K_a_ values for human milk oligosaccharide (HMO1 ­– HMO35) binding to LabA 26695.^a,b^

|  | Structure | Common name | K_a_  (10^3^ M^-1^) |
| --- | --- | --- | --- |
| HMO1 | α-L-Fuc-(1→2)-β-D-Gal-(1→4)-β-D-Glc | 2'-Fucosyllactose | 2.4±0.9 |
| HMO2 | β-D-Gal-(1→4)-[α-L-Fuc-(1→3)]-β-D-Glc | 3-Fucosyllactose | 1.7±0.6 |
| HMO3 | α-D-Neu5Ac-(2→3)-β-D-Gal-(1→4)-β-D-Glc | 3'-Sialyllactose | 2.0±0.5 |
| HMO4 | α-D-Neu5Ac-(2→6)-β-D-Gal-(1→4)-β-D-Glc | 6'-Sialyllactose | 3.3±1.1 |
| HMO5 | α-L-Fuc-(1→2)-β-D-Gal-(1→4)-[α-L-Fuc-(1→3)]-β-D-Glc | Difucosyllactose | 3.6±0.9 |
| HMO6 | β-D-Gal-(1→3)-β-D-GlcNAc-(1→3)-β-D-Gal-(1→4)-β-D-Glc | Lacto-N-tetraose | 2.2±0.5 |
| HMO7 | β-D-Gal-(1→4)-β-D-GlcNAc-(1→3)-β-D-Gal-(1→4)-β-D-Glc | Lacto-N-neotetraose | 2.9±0.4 |
| HMO8 | α- L-Fuc-(1→2)-β-D-Gal-(1→3)-β-D-GlcNAc-(1→3)-β-D-Gal-(1→4)-β-D-Glc | Lacto-N-fucopentaose I | 2.4±0.6 |
| HMO9 | β-D-Gal-(1→3)-[α-L-Fuc-(1→4)]-β-D-GlcNAc-(1→3)-β-D-Gal-(1→4)-β-D-Glc | Lacto-N-fucopentaose II | 2.4±0.6 |
| HMO10 | β-D-Gal-(1→4)-[α-L-Fuc-(1→3)]-β-D-GlcNAc-(1→3)-β-D-Gal-(1→4)-β-D-Glc | Lacto-N-fucopentaose III | 2.4±0.8 |
| HMO11 | β-D-Gal-(1→3)-β-D-GlcNAc-(1→3)-β-D-Gal-(1→4)-[α-L-Fuc-(1→3)]-β-D-Glc | Lacto-N-neofucopentaose V | 3.4±0.9 |
| HMO12 | β-D-Gal-(1→4)-β-D-GlcNAc-(1→3)-β-D-Gal-(1→4)[α-L-Fuc-(1→3)]-β-D-Glc | Lacto-N-neofucopentaose | 2.7±0.5 |
| HMO13 | α-D-Neu5Ac-(2→3)-β-D-Gal-(1→3)-β-D-GlcNAc-(1→3)-β-D-Gal-(1→4)-β-D-Glc | Sialyllacto-N-tetraose a | 5.0±1.8 |
| HMO14 | α-D-Neu5Ac-(2→6)-[β-D-Gal-(1→3)]-β-D-GlcNAc-(1→3)-β-D-Gal-(1→4)- β-D-Glc | Sialyllacto-N-tetraose b | 4.8±1.5 |
| HMO15 | α-D-Neu5Ac-(2→6)-β-D-Gal-(1→4)-β-D-GlcNAc-(1→3)-β-D-Gal-(1→4)-β-D-Glc | Sialyllacto-N-tetraose c | 2.8±0.7 |
| HMO16 | α-L-Fuc-(1→2)-β-D-Gal-(1→3)-[α-L-Fuc-(1→4)]-β-D-GlcNAc-(1→3)-β-D-Gal-(1→4)-β-D-Glc | Lacto-N-difucohexaose I | 2.6±0.8 |
| HMO17 | β-D-Gal-(1→3)-[α-L-Fuc-(1→4)]-β-D-GlcNAc-(1→3)-β-D-Gal-(1→4)-[α-L-Fuc-(1→3)]-β-D-Glc | Lacto-N-difucohexaose II | 3.0±0.6 |
| HMO18 | β-D-Gal-(1→4)-[α-L-Fuc-(1→3)]-β-D-GlcNAc-(1→3)-β-D-Gal-(1→4)-[α- L-Fuc-(1→3)]-β-D-Glc | Lacto-N-neodifucohexaose | 3.0±0.6 |
| HMO19 | β-D-Gal-(1→4)-β-D-GlcNAc-(1→3)-β-D-Gal-(1→4)-β-D-GlcNAc-(1→3)-β-D-Gal-(1→4)-β-D-Glc | Para Lacto-N-neohexaose | 2.3±0.5 |
| HMO20 | β-D-Gal-(1→4)-β-D-GlcNAc-(1→6)-[β-D-Gal-(1→4)-β-D-GlcNAc-(1→3)]-β-D-Gal-(1→4)-β-D-Glc | Lacto-N-neohexaose | 2.4±0.5 |
| HMO21 | α-D-Neu5Ac-(2→3)-β-D-Gal-(1→3)-[α-L-Fuc-(1→4)]-β-D-GlcNAc-(1→3)-β-D-Gal-(1→4)-β-D-Glc | Sialyl monofucosyllacto-N-tetraose | 3.2±0.5 |
| HMO22 | α-L-Fuc-(1→2)-β-D-Gal-(1→3)-[α-D-Neu5Ac-(2→6)]-β-D-GlcNAc-(1→3)-β-D-Gal-(1→4)-β-D-Glc | Sialyl-lacto-N-fucopentaose V | 2.7±0.5 |
| HMO23 | α-D-Neu5Ac-(2→3)-β-D-Gal-(1→3)-[α-D-Neu5Ac-(2→6)]-β-D-GlcNAc-(1→3)-β-D-Gal-(1→4)-β-D-Glc | Disialyllacto-N-tetraose | 2.7±0.6 |
| HMO24 | β-D-Gal-(1→4)-[α-L-Fuc-(1→3)]-β-D-GlcNAc-(1→6)-[α-L-Fuc-(1→2)-β-D-Gal-(1→3)-β-D-GlcNAc-(1→3)]-β-D-Gal-(1→4)-β-D-Glc | Difucosyllacto-N-hexaose a | 2.5±0.5 |
| HMO25 | β-D-Gal-(1→3)-[α-L-Fuc-(1→4)]-β-D-GlcNAc-(1→3)-β-D-Gal-(1→4)-[α-L-Fuc-(1→3)]-β-D-GlcNAc-(1→3)-β-D-Gal-(1→4)-β-D-Glc | Difucosyl-para-lacto-N-hexaose II | 2.6±0.5 |
| HMO26 | α-D-GalNAc-(1→3)-[α-L-Fuc-(1→2)]-β-D-Gal-(1→4)-β-D-Glc | Blood group A antigen tetraose type 5 | 2.5±0.5 |
| HMO27 | α-D-GalNAc-(1→3)-[α-L-Fuc-(1→2)]-β-D-Gal-(1→3)-β-D-GlcNAc(1→3)-β-D-Gal-(1→4)-β-D-Glc | Blood group A antigen hexaose type 1 | 2.5±0.5 |
| HMO28 | α-D-Neu5Ac-(2→3)-β-D-Gal-(1→4)-β-D-GlcNAc | 3'-Sialyl-N-acetyllactosamine | 2.4±0.6 |
| HMO29 | α-D-Neu5Ac-(2→6)-β-D-Gal-(1→4)-β-D-GlcNAc | 6'-Sialyl-N-acetyllactosamine | 4.4±1.1 |
| HMO30 | α-D-GalNAc-(1→3)-[α-L-Fuc-(1→2)]-β-D-Gal-(1→3)-[α-L-Fuc-(1→4)]-β-D-GlcNAc(1→3)-β-D-Gal-(1→4)-β-D-Glc | A-heptasaccharide | 2.5±0.5 |
| HMO31 | β-D-Gal-(1→4)-[α-L-Fuc-(1→3)]-β-D-GlcNAc-(1→6)-[β-D-Gal-(1→3)-β-D-GlcNAc-(1→3)]-β-D-Gal-(1→4)-β-D-Glc | Monofucosyllacto-N-hexaose III | 1.9±0.6 |
| HMO32 | β-D-Gal-(1→4)-[α-L-Fuc-(1→3)]-β-D-GlcNAc-(1→6)-[β-D-Gal-(1→3)-[α-L-Fuc-(1→4)]-β-D-GlcNAc-(1→3)]-β-D-Gal-(1→4)-β-D-Glc | Difucosyllacto-N-hexaose b | 1.7±0.5 |
| HMO33 | β-D-Gal-(1→4)-[α-L-Fuc-(1→3)]-β-D-GlcNAc-(1→3)-β-D-Gal-(1→4)-[α-L-Fuc-(1→3)]-D-GlcNAc-(1→3)-β-D-Gal-(1→4)-β-D-Glc | Difucosyl-para-lacto-N-neohexaose | 2.9±0.7 |
| HMO34 | β-D-Gal-(1→3)-β-D-GlcNAc-(1→3)-β-D-Gal-(1→4)-β-D-GlcNAc-(1→3)-β-D-Gal-(1→4)-β-D-Glc | Para Lacto-N-hexaose | 3.9±1.0 |
| HMO35 | β-D-Gal-(1→4)--[α-L-Fuc-(1→3)]-β-D-GlcNAc-(1→6)-[α-L-Fuc-(1→2)-β-D-Gal-(1→3)-[α-L-Fuc-(1→4)]-β-D-GlcNAc-(1→3)]-β-D-Gal-(1→4)-β-D-Glc | Trifucosyllacto-N-hexaose a | 3.2±0.6 |

a. Errors correspond to one standard deviation. b. Binding measurements were performed in 100 mM aqueous ammonium acetate solution (pH 6.8, 25 °C).
